# Properties and Multidisciplinary Applications of Zeolites and Mesoporous Materials

**DOI:** 10.3390/molecules31040735

**Published:** 2026-02-20

**Authors:** Łukasz Kuterasiński

**Affiliations:** Jerzy Haber Institute of Catalysis and Surface Chemistry, Polish Academy of Sciences, ul. Niezapominajek 8, 30-239 Kraków, Poland; lukasz.kuterasinski@ikifp.edu.pl

## 1. Introduction

Zeolites and mesoporous materials are important within the field of scientific research. The main applications of zeolites and mesoporous materials include their use as heterogeneous catalysts in the petrochemical industry and other industries; in biomass upgrading and the production of fine chemicals; in water softening and purification; in environmental pollution control; in gas separation, purification, and storage; in agriculture and aquaculture; in medicine and biotechnology; in the deactivation and immobilization of hazardous substances; and in nano-photonic and nano-sensor devices. The versatile applications of microporous zeolites and nanoporous materials in many industrial processes are facilitated by their unique properties, including their channel systems, uniform pore dimensions, shape selectivity, resistance to coke formation, and chemical, thermal, and hydrothermal stability. Furthermore, zeolites are a fascinating subject of research due to their crystalline structure, acidity, and well-defined pore system [1,2,3,4].

## 2. Water Purification

As industrialization accelerates, pollution from human activities has become pervasive, with water contamination posing particularly serious risks to both ecosystems and human health. Industries such as metal plating, metallurgy, oil refining, battery manufacturing, mining, textiles, and pharmaceuticals release waste that often ends up in groundwater, rivers, and lakes [5,6,7]. These wastes introduce a variety of pollutants into the environment, including heavy metals (lead, cadmium, copper, zinc, nickel), toxic anions (arsenate, chromate, fluoride, phosphate), organic dyes, and pharmaceutical residues, prompting the urgent need for effective methods of treating wastewater and restoring polluted water bodies. The wide-ranging nature of water contaminants makes treatment especially challenging [8]. Conventional approaches, including membrane filtration, chemical oxidation, solvent extraction, adsorption, coagulation, flocculation, and biological treatments, often have significant drawbacks. Many approaches rely on chemicals like chlorides, ammonia, permanganate, alum, sodium hydroxide, hydrochloric acid, ozone, or iron salts, requiring complex machinery, specialized engineering knowledge, and advanced infrastructure [9,10,11]. Treating each pollutant separately is usually impractical since traditional technologies may not fully remove toxins, phosphorus, nitrogen, and heavy metals [12]. In comparison, adsorption stands out for its simplicity, affordability, efficiency, and minimal secondary pollution. However, developing adsorbents with high capacities, fast kinetics, strong selectivities, and good recyclability remains a challenge [13].

Zeolites are especially suitable for treating wastewater due to their excellent thermal and chemical stability, high surface area, tunable pore structure, and abundance of ion-exchange sites, all of which help to efficiently remove toxic substances, including heavy metals, from water. Both natural and synthetic zeolites have been widely studied and have been shown to be highly effective in adsorbing water pollutants [14]. The International Zeolite Association’s Structural Committee recognizes more than 260 types of zeolites, among which zeolite A (also known as Linde Type A or LTA) stands out as the first commercially manufactured zeolite. It is commonly used for gas adsorption and ion exchange due to its low Si/Al ratio (SAR = 1), which provides it with a high adsorption capacity. LTA zeolites also feature three-dimensional channels that enable the rapid movement of ions for faster adsorption. Its straightforward, cost-effective synthesis makes zeolite an effective option for water purification applications [15,16].

Pan and colleagues (Contribution 1) recently reviewed advances in synthesizing LTA zeolites, emphasizing production processes that utilize natural materials and industrial solid wastes without relying on Structure-Directing Agents (SDAs). A low Si/Al ratio increases the number of ion-exchange sites and boosts the adsorption capacity of zeolites. Developing environmentally friendly, economical synthesis methods supports the growing need for sustainable solutions in water treatment. Their review covers the use of zeolite A and its derivatives for removing various water contaminants, providing benchmark results to guide future research. For metal cation adsorption, they summarize the order of selectivity among different cations based on experimental data and prior studies, offering useful insights into ion-exchange mechanisms.

Prioritizing the development of low-cost, eco-friendly zeolite A adsorbents made using natural sources and industrial waste—while improving their adsorption performance—is crucial. The established ion-exchange selectivity can help optimize treatment processes for both industrial and domestic effluents and even enable the selective recovery of valuable metals, supporting circular economy practices and sustainability goals. Significant progress has been made in creating zeolite A from solid waste for use in practical applications. Setting up systems to synthesize zeolite A on-site at waste generation locations or near polluted waters could further streamline the process, converting waste directly into zeolite A for immediate use in water treatment. This would reduce the transportation needs for raw materials and adsorbents, cut costs, and enhance sustainability overall.

In turn, Zahid and co-researchers (Contribution 2) studied how acid-treated natural clinoptilolite and mordenite zeolites from Almaty could improve adsorption for drinking water purification. Acid modification partially removed aluminum, increasing surface defects and micropores, which improved the ion-exchange capacity and selectivity for heavy metals. Modified clinoptilolite achieved a removal efficiency of 94% for Pb^2+^, 86% for Cd^2+^, and 84% for As^3+^, while mordenite reached efficiencies of 95%, 90%, and 87%, respectively. The enhanced performance was attributed to the increased surface area and active ion-exchange sites, as confirmed through Brunauer–Emmett–Teller (BET) analysis. Notably, these modified zeolites retained over 80% of their adsorption capacity after five cycles, demonstrating a high reusability. Compared to conventional purification methods like activated carbon and membrane filtration, acid-modified zeolites offer cost-effective, environmentally friendly alternatives that produce negligible secondary waste. These findings indicate that treated clinoptilolite and mordenite have a significant potential for sustainable heavy metal removal in drinking water systems.

## 3. Waste Recycling and Dye Removal in the Textile Industry Using Li-Functionalized Nanoparticle Zeolites

The textile industry plays a vital role in many economies but is also a major source of water pollution, primarily due to the discharge of large volumes of wastewater. Textile dyeing releases harmful organic compounds like dyes, which are non-degradable and highly carcinogenic, posing serious threats to human health. Dyes also increase water turbidity, reducing the dissolved oxygen for aquatic life, and their bioaccumulation through the food chain raises further health risks, especially from contaminated seafood. Removing these dyes from textile wastewater is crucial, and strict regulations are needed to protect natural water bodies [17,18].

Methylene blue (MB), a sulfur-containing heterocyclic aromatic dye classified as an azo dye, is widely used in textile manufacturing for cotton, silk, and wool. However, MB exposure can cause adverse health effects, including breathing problems if inhaled and symptoms like nausea, diarrhea, vomiting, itching, and gastritis if ingested, especially at high concentrations [19,20]. Various methods have been studied for removing organic pollutants from water, including adsorption, ion exchange, photocatalysis, chemical oxidation, and coagulation. Recently, catalytic degradation and adsorption have gained attention for their effectiveness. The materials used for MB adsorption range from activated carbon and magnetic zeolites derived from fly ash to graphene oxide-modified zeolites [21].

Adsorption stands out as one of the most efficient and practical methods for pollutant removal, especially for persistent dyes like methylene blue. The mechanisms of pollutant removal include physical and chemical adsorption, as well as ion exchanges. However, challenges remain in developing adsorbents with a high adsorption capacity [22]. Current research seeks low-cost and improved adsorbents sourced from natural minerals or agricultural and industrial wastes. Zeolites in particular have been extensively studied because of their excellent cation-exchange abilities, which make them suitable for removing cationic dyes like MB [23,24]. Modifying zeolites with metals such as lithium (Li^+^) can enhance their structural and adsorption properties, allowing for tailored pore selectivity and an easier diffusion of pollutants. Lithium has shown promise in various catalytic systems, improving attributes like the surface area and charge separation efficiency for different chemical reactions [25,26].

Guaya et al. (Contribution 3) explored the incorporation of lithium into a zeolitic sample called MT-ZLSH, synthesized from mining tailings and comprising Linde Type A (LTA) and sodalite–hydroxysodalite (ZLSH). The addition of lithium-altered functional groups and surface properties led to nanometric phase formation and an improved MB adsorption efficiency, even within the pH ranges typical for real wastewater. The adsorption process involved both physical and chemical mechanisms, including ion exchange, electrostatic attraction, and hydrogen bonding. Thermodynamic studies confirmed the exothermic nature of MB adsorption, and kinetic models suggested distinct stages, with intraparticle diffusion as the limiting step. Photocatalytic tests showed that MT-ZLSH-Li+ could degrade 77% of MB in 180 min, though its performance dropped in subsequent cycles due to the reduced active centers and the accumulation of byproducts. Compared to other catalysts, MT-ZLSH-Li+ performed competitively, though it required more time, likely due to its limited surface area and active phase content. While it shows potential for wastewater treatment, especially for adsorption, further improvements are necessary to achieve a higher efficiency and stability. Additionally, due to the high solubility of lithium compounds, the material may be better suited for non-aqueous applications.

## 4. Cu-Containing Zeolites as Catalysts for the Manufacture of Biaryls

A biaryl is a molecular structure formed by two aromatic rings joined by a carbon–carbon bond. These structures are common in biologically active compounds and natural products and have become increasingly important as drug candidates in medicinal chemistry [27].

**Figure 1:**
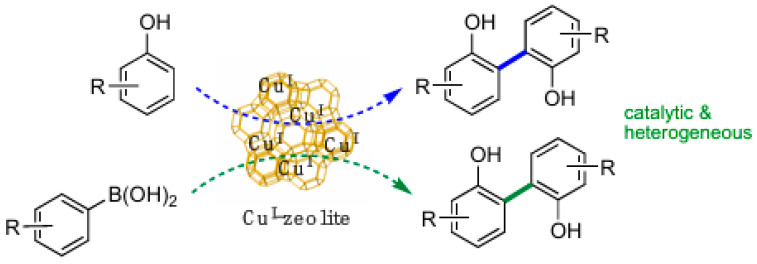
The methodology for biaryl synthesis utilizing a Cu^I^-supported zeolite catalyst (Contribution 4).

Recognizing the significance of biaryls in natural products, pharmaceuticals, agrochemicals, dyes, and organic electronics, Di et al. (Contribution 4) developed an environmentally friendly method for synthesizing biaryls. This approach utilizes copper(I)-exchanged zeolite catalysts, which are both easy to prepare and cost-effective (Figure 1). Specifically, copper-containing ultrastabilized zeolite Y (Cu^I^-USY) efficiently catalyzes the direct homocoupling of phenols or aryl boronic acids under straightforward, practical conditions.

For the oxidative homocoupling of phenols, Cu^I^-USY operates effectively in either warm methanol or water and can be used conveniently in air, achieving good to high yields. A small quantity of Cs_2_CO_3_ is needed when using methanol, but the reaction in water requires none. The homocoupling of aryl boronic acids is best conducted in warm methanol without any additives. These gentle reaction conditions accommodate various functional groups, resulting in numerous substituted (hetero)biaryls—28 examples are demonstrated. The Cu^I^-USY catalyst, being heterogeneous, is easy to recover and reuse.

Additionally, the homocoupling of vinyl boronic acids was integrated into a Diels–Alder reaction, even within a one-pot process. This innovation provides access to highly functionalized cyclohexenes.

## 5. Cu-Zeolites as Modifiers of ANFO-Based Energetic Materials

A comprehensive review of the literature concerning industrial applications of zeolites indicates that utilizing zeolites as modifiers for energetic materials represents a novel aspect of contemporary research. Ammonium Nitrate Fuel Oil (ANFO) is one of the most widely employed energetic materials in civil sectors—particularly in open-pit mining and large-scale earthworks—owing to its cost-effective production and favorable energetic properties [28]. The attractiveness of zeolites as ANFO modifiers stems from their chemical composition. For example, the inclusion of aluminum, organic templates, or introduced metallic guest atoms at extra-framework locations in zeolites can serve as combustible components and sensitizers (analogous to fuel oil), thereby influencing energetic parameters such as pressure, temperature, heat output, strength, and decomposition velocity [29,30,31]. Conversely, the silica–oxygen (Si–O) framework in zeolites acts as an inert phase, capable of absorbing energy and thus mitigating the intensity and heat of decomposition, among other effects [32,33].

Regardless of whether the additive is inert or chemically active, its integration will inevitably affect the material density and oxygen balance, resulting in modifications to the energetic characteristics—namely the energy, heat, and decomposition pressure—which subsequently alter the fume composition. Notably, combustible elements utilized in the formulation of ANFO-based materials include not only organic compounds (e.g., fuel oil, activated carbon, charcoal, hydrocarbons, and derivatives) but also inorganic substances such as metallic powders, salts, and oxides [29,30,31]. The dual functionality of zeolite constituents—wherein certain elements function as combustible agents and others function as inert phases—renders zeolites particularly compelling as multi-purpose modifiers within the energetic materials industry.

Given the established use of copper zeolites as DeNO_x_ catalysts, integrating Cu-containing zeolites as additives in ANFO formulations can reduce the toxic emissions generated during decomposition [34,35,36]. In this context, Kuterasiński et al. (Contribution 5) investigated the incorporation of FAU-type Cu-containing zeolites into eco-friendly ANFO materials. All physicochemical properties of these zeolite-modified ANFO samples were systematically compared with those of unmodified controls.

Thermodynamic modeling and experimental analyses demonstrated that adding FAU-type Cu-containing zeolites to ANFO enhances key decomposition parameters—including the pressure, strength, temperature, heat release, and reaction velocity—while significantly reducing the total post-decomposition fume emissions, which is highly beneficial from an environmental standpoint. The observed energetic performance was closely linked to the surface characteristics and, to a lesser extent, the structure and thermal properties of the zeolite-modified samples. Enhanced energetic outcomes correlated positively with increasing concentrations of Cu-faujasite, particularly when copper occupied monovalent cationic exchange sites.

For Cu-free FAU-type zeolites, the effect of the Si/Al ratio on the performance of ANFO composites was examined. Under anhydrous conditions, zeolites with lower Si/Al ratios exhibited a superior enhancement of energetic properties, attributable to their higher aluminum content, which acts as a supplementary fuel. Under hydrated conditions, however, the increased hydrophilicity of low-Si/Al-ratio zeolites diminished the energetic parameters. Importantly, both the energetic evaluations and analysis of post-decomposition emissions suggest that the developed ANFO-based materials hold promise as low-emission energy sources suitable for various industrial and individual applications.

## 6. Ni-Zr/Zeolite Catalysts for the Hydrocracking of AlgalOil

Decades of social progress, coupled with the expansion of industry, transportation, and population, have continually increased global energy demands. Relying on fossil fuels to meet these needs is problematic—not only are these resources finite, but burning them releases harmful gases like carbon dioxide (CO_2_), carbon monoxide (CO), nitrous oxide, nitrogen oxides (NO_x_), and sulfur oxides (SO_x_), all of which damage the environment. The depletion and non-renewable nature of oil and natural gas, their negative ecological impact, and the risk of energy shortages underscore the urgency to find alternative renewable fuels. One promising avenue involves converting vegetable and algal oils into biofuels—both animal fats and plant-based oils deliver high conversion rates [37,38].

Modern biofuel production is shifting toward third-generation fuels, particularly those made from algal biomass or oils. Algae offer several key advantages over traditional crops used for biofuels: they produce higher oil yields, require minimal resources, and can be cultivated without occupying farmland. As fast-growing organisms, algae transform sunlight, water, and carbon dioxide into biomass through photosynthesis. Additionally, algae can utilize nutrients from municipal and agricultural wastewater, which are often detrimental to conventional crops. Algae also excel at sequestering CO_2_ thanks to their high photosynthetic efficiency, which further accelerates their growth. Beyond being a rich source for oil extraction and hydrocracking, algae can also serve as a raw material for biogas, biodiesel, bioethanol, and other commercially valuable products [39,40,41].

One common technique for producing alternative biofuels is catalytic hydroprocessing, which includes hydrotreating and hydrocracking. Hydrocracking, in particular, uses bifunctional heterogeneous catalysts to break down large molecules—such as triglycerides in oils—into much smaller hydrocarbons (mainly C_15_–C_18_) under high temperatures (260–425 °C) and pressure (35–200 bar) in a hydrogen-rich atmosphere. This process generates alkanes, olefins, and aromatic hydrocarbons, making it a versatile method for creating hydroprocessed kerosene, green kerosene, various synthetic paraffinic kerosenes, and bio-hydrogenated kerosene, which can be used as alternative diesel or jet fuels [42,43,44]. Catalysts in this process often feature noble metals like platinum or combinations of nickel with molybdenum or tungsten; these materials are valued for their efficiency and selectivity in catalysis. Supporting these active metals on porous surfaces improves their stability. Nickel-based catalysts, in particular, have gained prominence because they provide an excellent performance at a lower cost than noble metals [45,46,47].

For applications that demand a strong thermal stability, zeolites are commonly used as crystalline catalysts. Recent research has explored mesoporous and zeolitic materials enriched with zirconium (Zr), revealing that Zr enhances catalytic properties. These Zr-containing porous materials exhibit both acidic and hydrogenating characteristics, though their use in hydrocracking reactions remains understudied [48]. To address this gap, Szkudlarek et al. (Contribution 6) prepared catalysts based on BEA and ZSM-5 zeolites, as well as classical alumina oxide, augmented with Zr species specifically for hydrocracking algal oil. In their study, Szkudlarek and colleagues examined the hydrocracking of *Spirulina platensis* algae oil using bi-component nickel–zirconia catalysts supported on BEA, ZSM-5, or Al_2_O_3_ and prepared through wet co-impregnation. They analyzed both the effect of catalyst support and how reductions in temperature influenced catalytic activity and product selectivity. Their results showed that Ni-Zr systems on zeolite supports achieved high oil conversion rates, though increasing the reduction temperature from 500 °C to 600 °C and 700 °C resulted in lower conversions for BEA-supported catalysts. The hydrocracking yielded hydrocarbons ranging from C_7_ to C_33_ with zeolite catalysts and up to C_36_ with Al_2_O_3_ systems, mostly within the gasoil fraction (C_14_–C_22_). The best performance was observed with a 5%Ni-5%Zr/BEA catalyst reduced at 600 °C, delivering an algal oil conversion rate of 94.0%. The variation in catalytic efficiency was attributed to differences in the surface area and the acidity of the catalysts, which depended on the support and reduction conditions.

## 7. Mesoporous Materials in Gene Therapy

CRISPR-Cas9 technology (*Clustered Regularly Interspaced Short Palindromic Repeats*, with Cas9 as a protein that works alongside CRISPR) is one of the most groundbreaking genome editing tools for tackling genetic disorders [49]. Despite its power, precision, and efficiency, challenges like delivery efficiency and targeting specificity still limit its clinical use. In their review, Lee et al. (Contribution 7) offer an in-depth analysis of the structural characteristics, benefits, and possible uses of different non-viral and stimuli-responsive delivery systems. They highlight recent advances, showing how these systems may help overcome key limitations and lead to further progress in CRISPR-Cas9 therapeutics.

### 7.1. Structure and Characterizations of Mesoporous Silica Nanoparticles 

**Figure 2:**
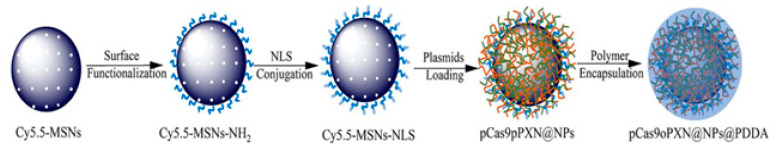
Scheme of functionalized MSN-based nanocarriers for CRISPR-Cas9 delivery. Adapted from [51] and based on CC-BY4.0 license.

Mesoporous silica nanoparticles (MSNs) are emerging as excellent tools for transporting therapeutic genetic materials, including CRISPR-Cas9 systems. Their distinctive features—such as a large surface area, adjustable pore and particle sizes, and customizable surfaces—enable them to carry and deliver sizable genetic material efficiently, making them well suited for gene therapy applications [50]. By attaching ligands, cationic polymers, or peptides to MSNs (see Figure 2), their ability to enter cells, along with their transfection rates, can be improved, broadening their usefulness [51].

Surface modification is crucial for the efficiency of MSNs in delivering genes. Untreated MSNs have negatively charged silanol groups, which repel similarly charged nucleic acids. To address this, researchers often introduce positive charges through amination or coating with cationic polymers, such as polyethylenimine (PEI). PEI-coated MSNs enhance the attraction to nucleic acids, ensuring that DNA is compacted and protected, while also helping it escape from endosomes. This results in a better gene expression and lower toxicity, positioning these MSNs as strong candidates for CRISPR-Cas9 delivery [52].

Another important feature of MSNs is the ability to tailor pore size, which helps control how genetic therapies are loaded and released. Larger pores can hold bigger molecules like CRISPR-Cas9, but they risk releasing their contents too quickly. To prevent this, non-covalent binding methods have been used; for example, nickel-functionalized MSNs carrying His-tagged β4 proteasome subunits (29 kDa) inside 25–30 nm pores successfully delivered these proteins and broke down excess tau protein in HEK293 cells—a critical step for treating Alzheimer’s disease. This MSN-based approach was more effective at reducing tau levels than standard proteasomes, highlighting its promise for treating disorders caused by misfolded proteins [53,54]. MSNs also stand out for being structurally stable and biodegradable within living organisms. Their breakdown products, like silicic acid, are safe and do not build up in the body. Still, damage to their surfaces—especially in watery environments with amines—can be a problem.

Techniques such as modifying MSNs with 3-aminopropyl(triethoxy)silane (APTES) or adding zwitterionic groups help prevent deterioration, contamination, and clumping in biological systems. Overall, these properties make MSNs highly suitable for nanomedicine, as they reduce toxicity and immune reactions while maintaining strength and reliability for therapeutic delivery [55,56].

### 7.2. Advantages of Mesoporous Silica Nanoparticles (MSNs) in CRISPR-Cas9 Delivery

The surface functionalization of mesoporous silica nanoparticles (MSNs) markedly improves their targeting capabilities, thereby addressing one of the principal challenges associated with CRISPR-Cas9 systems—namely, their off-target effects [57]. An aptamer-based surface modification is a prominent strategy in this regard. Aptamers are short, single-stranded oligonucleotides (2–80 nucleotides) that exhibit a high specificity and affinity for designated molecular targets. When conjugated to MSNs, these aptamers enable the efficient internalization of therapeutic agents into target cells, facilitating tissue-specific delivery. This method not only mitigates off-target activity but also enhances efficiency, which is typically limited in non-viral delivery approaches. For instance, Yang et al. introduced an MSN-PEM-based aptamer nanocarrier employing DNA aptamer gc8, which achieved a notable cell recognition and drug delivery performance in cancer cells [58]. Such findings underscore the dual benefits of MSN surface engineering: elevated gene delivery efficacy and the enhanced applicability of CRISPR-Cas9 therapeutics via targeted delivery and controlled release mechanisms.

MSNs are increasingly recognized as versatile platforms for the concurrent delivery of CRISPR-Cas9 components alongside small-molecule drugs—a strategy that holds significant potential for stimuli-responsive nanoformulations addressing complex diseases. Fernández et al. devised a multifunctional MSN platform capable of co-delivering the anti-inflammatory agent VX-765 and CRISPR-Cas9, successfully editing the Gasdermin D gene, diminishing inflammation-driven cell death, and amplifying anti-inflammatory outcomes. These studies illustrate the capacity of MSNs for co-delivery applications, maximizing therapeutic efficacy through the integration of gene editing and pharmacological interventions [59].

The porous architecture of MSNs also enables the creation of theranostic systems that unite therapeutic and diagnostic functions. This “theranostics” paradigm merges treatment modalities with advanced imaging techniques, such as MRI, photoacoustic imaging, near-infrared fluorescence (NIRF), PET, SPECT, and CT (X-ray), thus facilitating precise lesion localization while administering therapeutics. MSN-based theranostic platforms can be tailored to leverage the strengths of various therapeutic and imaging agents, allowing for a patient-specific approach within personalized medicine frameworks. In particular, MSN-based gene therapies show considerable promise for image-guided cancer diagnosis and treatment, highlighting their role in advancing precision medicine [60,61].

Nevertheless, several important challenges remain before MSN-mediated CRISPR-Cas9 delivery systems can be translated into clinical practice. Critical considerations include ensuring long-term stability, appropriate in vivo distribution, and the biodegradability of MSNs. Additionally, comprehensive validation using robust preclinical models and clear regulatory protocols will be vital for facilitating the progression of these platforms from research to clinical applications [62].

## 8. Conclusions

The studies featured in this Special Issue collectively highlight the extensive range of applications for zeolites and mesoporous materials, encompassing areas such as catalysis, drinking water purification, textile industry waste recycling, biaryl synthesis for multiple industrial uses, the modification of energetic materials, and the delivery of genetic material in CRISPR-Cas9 gene therapy. These works exemplify the ongoing research into the vast potential for further applications of zeolites and mesoporous materials, with a focus on sustainability, innovation, and interdisciplinary collaboration. Collectively, they underscore that research on zeolites and mesoporous materials remains an active and dynamic field, offering significant promise for the advancement of technologies that address contemporary multidisciplinary challenges.

## Data Availability

Not applicable.
